# Supplementary material for: A computational approach to calculate the heat of transport of aqueous solutions
Source: Sci Rep. 2017 Mar 21;7:44833. doi: 10.1038/srep44833 (PMC5359663; doi:10.1038/srep44833)
Supplement: Supplementary Information [file srep44833-s1.pdf]

Supplementary Information: A computational approach to calculate the heat of transport of aqueous solutions

Silvia Di Lecce[1], *Tim Albrecht*[1], *and Fernando Bresme*[1,*]

Supporting Information:

$T\ [K]$	$b\ [kg\ mol^{-1}]$	$S_T \times 10^3\ [K^{-1}]$
240	0.934 ± 0.034	-3.57 ± 0.28
	1.406 ± 0.038	-5.42 ± 0.31
	1.896 ± 0.046	-5.93 ± 0.14
	2.385 ± 0.032	-7.22 ± 0.38
	2.881 ± 0.029	-5.42 ± 0.40
	3.460 ± 0.086	-3.98 ± 0.86
	4.065 ± 0.082	-2.69 ± 0.67
	4.596 ± 0.054	-3.03 ± 0.09
	5.234 ± 0.054	-2.61 ± 0.04
270	1.013 ± 0.029	-1.878 ± 0.25
	1.565 ± 0.029	-2.08 ± 0.26
	2.102 ± 0.038	-1.69 ± 0.30
	2.680 ± 0.034	-1.73 ± 0.10
	3.213 ± 0.042	-2.28 ± 0.08
	3.775 ± 0.042	-2.05 ± 0.18
	4.347 ± 0.038	-1.84 ± 0.17
	4.939 ± 0.049	-1.89 ± 0.04
	5.580 ± 0.053	-1.75 ± 0.02
290	1.040 ± 0.025	-0.74 ± 0.23
	1.606 ± 0.023	-0.62 ± 0.12
	2.146 ± 0.028	-0.52 ± 0.17
	2.735 ± 0.030	-0.50 ± 0.08
	3.321 ± 0.035	-1.09 ± 0.12
	3.898 ± 0.036	-1.21 ± 0.02
	4.487 ± 0.032	-1.34 ± 0.02
	5.101 ± 0.048	-1.35 ± 0.02
	5.755 ± 0.053	-1.33 ± 0.01

Table S1. Soret coefficients obtained in this work as a function of salt concentration and temperature. The NEMD simulations were performed using the system equilibrated at an average pressure of 600 bar.

T [K]	b [$kg\ mol^{-1}$]	$S_T \times 10^3$ [K^{-1}]
240	0.938 ± 0.030	-3.39 ± 0.24
	2.351 ± 0.027	-5.79 ± 0.49
	3.965 ± 0.076	-3.71 ± 0.34
270	1.046 ± 0.030	-2.04 ± 0.22
	2.736 ± 0.044	-2.31 ± 0.13
	4.460 ± 0.032	-2.13 ± 0.08
290	1.038 ± 0.022	-0.71 ± 0.20
	2.728 ± 0.034	-1.12 ± 0.11
	4.484 ± 0.043	-1.50 ± 0.09

Table S2. Same as Table S1 for average pressure 100 bar.

	b [$kg\ mol^{-1}$]	N_{pair}	N_{H_2O}	V [nm^3]	μ_{id} [$kJ\ mol^{-1}$]	μ_{ex} [$kJ\ mol^{-1}$]
Li^+	1.100	16	808	24.20	−6.62	-480.47 ± 0.45
	2.075	31	830	25.53	−5.42	-478.46 ± 0.38
	2.992	43	798	25.09	−4.71	-477.52 ± 0.65
	3.916	54	766	24.69	−4.23	-476.72 ± 0.57
Cl^-	1.100	16	808	24.27	−6.63	-369.98 ± 0.85
	2.075	31	830	25.56	−5.41	-370.21 ± 0.96
	2.994	43	798	25.18	−4.72	-372.30 ± 0.87
	3.916	54	766	24.77	−4.23	-371.17 ± 0.96

Table S3. Excess chemical potential μ_{ex} and ideal term μ_{id} for the Li^+ and Cl^- as a function of salt concentration, at 240 K and 600 bar.

	b [$kg\ mol^{-1}$]	N_{pair}	N_{H_2O}	V [nm^3]	μ_{id} [$kJ\ mol^{-1}$]	μ_{ex} [$kJ\ mol^{-1}$]
Li^+	1.100	16	808	24.47	−7.57	-474.61 ± 0.15
	2.075	31	830	25.80	−6.10	-473.02 ± 0.17
	2.994	43	798	25.42	−5.27	-471.75 ± 0.19
	3.916	54	766	25.01	−4.68	-470.69 ± 0.19
Cl^-	1.100	16	808	24.56	−7.58	-366.82 ± 0.68
	2.075	31	830	25.88	−6.10	-367.43 ± 0.56
	2.994	43	798	25.50	−5.28	-367.61 ± 0.64
	3.916	54	766	25.09	−4.69	-367.80 ± 0.49

Table S4. Same as Table S3 for 290 K.

T $[K]$	parameter	LiCl	Li^+
240	A $[mol^{\frac{1}{2}}\ kg^{-\frac{1}{2}}]$	-21.9118	-15.50
	B $[mol^{\frac{1}{2}}\ kg^{-\frac{1}{2}}]$	0.5663	0.28
	β $[kg\ mol^{-1}]$	-8.9211	-10.50
	C $[kg^2\ mol^{-2}]$	3.9684	3.45
	D $[kg^3\ mol^{-3}]$	-0.2708	-0.40
	K $[kJ\ mol^{-1}]$	-868	-490
290	A $[mol^{\frac{1}{2}}\ kg^{-\frac{1}{2}}]$	-12.91180	-10.59149
	B $[mol^{\frac{1}{2}}\ kg^{-\frac{1}{2}}]$	0.56629	0.64024
	β $[kg\ mol^{-1}]$	-6.92105	-4.64390
	C $[kg^2\ mol^{-2}]$	2.96837	1.41250
	D $[kg^3\ mol^{-3}]$	-0.27076	-0.14137
	K $[kJ\ mol^{-1}]$	-860	-485

Table S5. Fitting parameters obtained with equation (5) in the main text.
